# Deciphering Innate Immune Cell-Tumor Microenvironment Crosstalk at a Single-Cell Level

**DOI:** 10.3389/fcell.2022.803947

**Published:** 2022-05-13

**Authors:** Ryohichi Sugimura, Yiming Chao

**Affiliations:** Centre for Translational Stem Cell Biology, School of Biomedical Sciences, The University of Hong Kong, Pokfulam, Hong Kong SAR, China

**Keywords:** ScRNA-seq, tumor microenvironment, innate immune cell, data mining, coding

## Abstract

The tumor microenvironment encompasses various innate immune cells which regulate tumor progression. Exploiting innate immune cells is a new frontier of cancer immunotherapy. However, the classical surface markers for cell-type classification cannot always well-conclude the phenotype, which will further hinge our understanding. The innate immune cells include dendritic cells, monocytes/macrophages, natural killer cells, and innate lymphoid cells. They play important roles in tumor growth and survival, in some cases promoting cancer, in other cases negating cancer. The precise characterization of innate immune cells at the single-cell level will boost the potential of cancer immunotherapy. With the development of single-cell RNA sequencing technology, the transcriptome of each cell in the tumor microenvironment can be dissected at a single-cell level, which paves a way for a better understanding of the cell type and its functions. Here, we summarize the subtypes and functions of innate immune cells in the tumor microenvironment based on recent literature on single-cell technology. We provide updates on recent achievements and prospects for how to exploit novel functions of tumor-associated innate immune cells and target them for cancer immunotherapy.

## Introduction

### Overview of the Tumor Microenvironment

**FIGURE 1:**
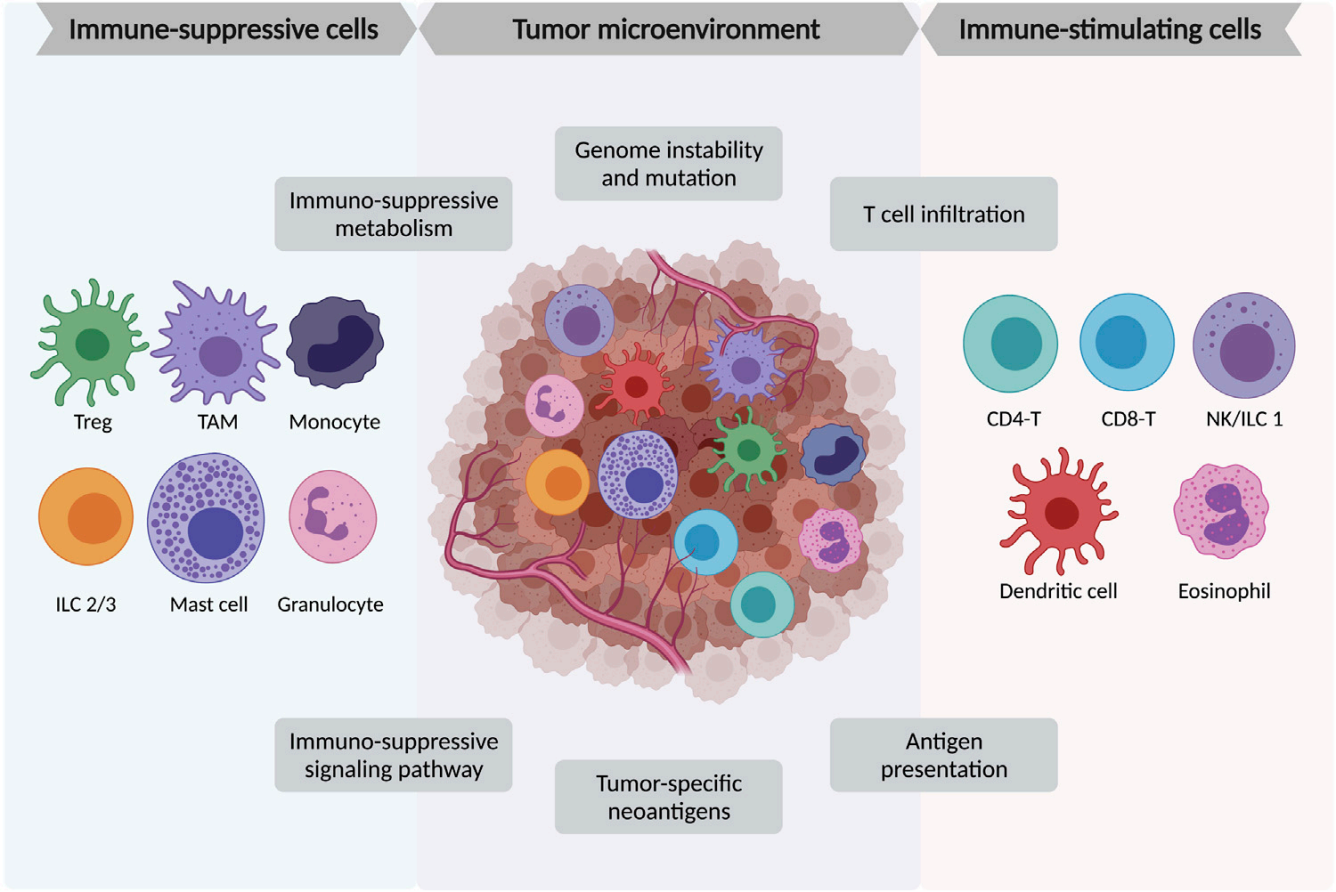
Major immune cell components in tumor micro-environment (TME). TME is a complex structure containing heterogeneous tumor cells, blood vessels, various immune cells, stromal cells, and the extracellular matrix. Among the immune cells, the immune-suppressive cells include FoxP3+ regulatory T cells (Treg), tumor-associated macrophages (TAMs), monocytes, innate lymphoid cells type 2 and 3 (ILC2/3), mast cells, and granulocytes. The immune-stimulating cells include tumor-infiltrating CD4+ and CD8+ lymphocytes, natural killer (NK) cells/innate lymphoid cells type 1 (ILC1), dendritic cells, and eosinophils.

The tumor microenvironment (TME) is a complicated structure containing diverse immune cells, stromal cells (including fibroblasts, vascular networks, mesenchymal stromal cells, pericytes, and adipocytes), extracellular matrix, and multiple signaling molecules. The immune cells consist of innate immune cells including some myeloid suppressive cells and innate lymphoid cells, and adaptive immune cells like T and B lymphocytes. The stromal cells, including CAFs (cancer-associated fibroblasts) and adipocytes, also play as immunomodulators in TME (Gunaydin, 2021; Wu et al., 2021). Other non-cellular components including the growth factors, cytokines, small regulatory RNAs, and metabolites released from the tumor or immune cells could further remodel the TME (Patel et al., 2018) (Figure 1).

Accumulating evidence has revealed that the relevant cellular and non-cellular components within TME can regulate the tumor initiation, growth, metastasis, and immune response to cancer therapies (Jin and Jin, 2020). Tumors are initiated due to complex biological factors, leading to hyperplasia beyond control. Within the tumor progression or invasion, TME gradually becomes hypoxic, and the acidosis structure is based on tumor cell metabolism. At the same time, various immune cells also play different roles in immune response, recognition, cytotoxicity, and interaction with each other. Understanding the role of each player in TME, especially at a single-cell level, can help us better establish the knowledge network and design effective therapeutic strategies. In this review, we will focus on the role of innate immune cells in TME. With the recent public available single-cell datasets, we will discuss their novel subtypes, the involved immune response or pathways in the tumor, and potential predictive values for therapeutic targets.

## Innate Immune System and Cell Components

The innate immune system is the first barrel of our immunology compared with the adaptive immune system. The major functions include immune cell recruitment by cytokines, complement cascade activation, external substances identification and removal, adaptive immune system initiation, and an overall physical and chemical barrier of our body. Among these, the innate immune cells consist of myeloid cells, like dendritic cells (DCs), monocytes, macrophages, neutrophils, as well as the lymphoid cells, such as natural killer cells (NKs) and innate lymphoid cells (ILCs).

DCs are one of the most strengthened antigen-presenting cells in the mammalian immune system, bridging the activation of innate and adaptive immunology. DCs are often located in the tissues directly facing the external environment, like the skin, nose, lung, digestive system, and bloodstream. Upon the antigen-activated, they migrate to lymph nodes and launch and regulate the adaptive immune system through interaction with T and B lymphocytes. In TME, DCs infiltrate tumors and present the naïve T cells with processed tumor-derived antigens for T cell functioning. The infiltration of DCs can be a predictive marker for survival evaluation (Melaiu et al., 2020).

Monocytes are a large proportion of leukocytes and can differentiate into macrophages upon stimulation. They take part in the innate immune system as well as regulate the adaptive immune system. In cancers, monocytes play roles in both anti- and pro-tumor immunity, through cytokine secretion, phagocytosis, angiogenesis promotion. Phenotypical changes of monocytes in peripheral blood can be used for diagnosis, prediction, and prognosis (Hamm et al., 2016; Kiss et al., 2020).

Macrophages are another type of leukocyte capable of phagocytosis, antigen presentation, and anti-inflammatory function. The tissue-resident macrophages exist in nearly all tissue in our body and exhibit tissue-specific functions (Davies et al., 2013). Along with monocytes, they are composite for the mononuclear phagocyte system. In TME, macrophages are a double-edged sword, as they can directly phagocytose the tumor and remodel TME, but at the same time, they can also promote angiogenesis for tumor progression. Based on these properties, macrophages have opened a new avenue for cancer immunotherapy (Duan and Luo, 2021).

Neutrophils are the most abundant granulocytes accounting for 50–70% of the human bloodstream possessing high cell heterogeneity in homeostasis and infection (Xie et al., 2020). They are the marker of acute inflammation and are recruited to the injury sites for phagocytosis. This process is called chemotaxis, leading to the neutrophils’ migration toward sites of infection or inflammation, directed by cytokines and the complement system. In the tumor, neutrophils can be polarized into anti-tumor (N1) or pro-tumor (N2) phenotypes (Shaul and Fridlender, 2019).

NK cells are cytotoxic lymphocytes belonging to the innate immune system. It is increasingly accepted as a type of innate lymphoid cell as they develop from the common lymphoid progenitor. Upon pathogen infection or tumor formation, NK cells can rapidly respond, recognize, and attack the stressed cells without antibody or MHC recognition, enabling a fast immune reaction. In cancer, NK cells also exhibit potential for immunotherapy and prognosis (Melaiu et al., 2020; Liu et al., 2021).

ILCs are another heterogeneous lymphoid population in the innate immune system, which are mainly tissue-resident cells and rarely show up in blood circulation. They mainly secrete cytokines to regulate innate and adaptive immunity. Many of ILCs function similarly to T cells, which are likely to be the innate counterparts of T cells (Panda and Colonna, 2019). In cancer, ILCs exhibit diverse functions, with various clinical predictive values listed in this review (Bruchard and Ghiringhelli, 2019).

## How Single-Cell Technologies Facilitate Our Understanding of Innate Immune Cells in Tumor Microenvironment

**FIGURE 2:**
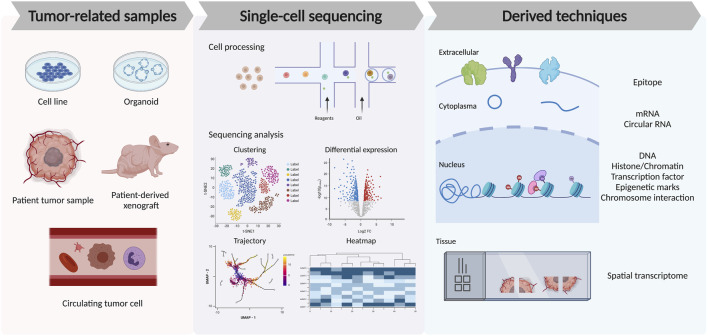
Recent advances in single-cell technologies to analyze tumor microenvironment. The left panel shows different tumor-related sample types used for single-cell TME studies. The middle panel shows the cell process of droplet-based single-cell sequencing technology and the downstream analysis from sequencing data, including the clustering and cell-type annotation, differential gene expression among samples or cell types, trajectory analysis of the cell lineage, and heatmap for gene dynamics. The right panel shows the recent derived techniques from single-cell sequencing, which allows the detection of the single-cell epitope, mRNA, circular RNA, DNA, epigenetic modification, chromosome interaction, and spatial information among the tissue.

In cancer studies, multiple sample types have been sequenced for different purposes. For example, tumor cell lines, tumor spheroids or organoids, patient tumor resections, patient-derived xenografts, and circulating tumor cells (CTCs) from liquid biopsies are common samples for cancer research. Normally, for single-cell RNA-seq, fresh samples are preferred and followed by standard sample preparation protocol, including tissue dissociation, filtration, and RBC lysis. Dead cells and doublets removal are usually required and can be achieved by flow cytometry with optional specific cell sorting like immune cell enrichment and filtration. After this, the single-cell suspension will be sent for cell encapsulation, library construction, and in-depth sequencing. For processing frozen or hard-to-dissociate tissues, like bone, adipose, and liver, single-nucleus RNA-seq (snRNA-seq) are required for better preservation of the samples and capture of the nuclear transcripts (Slyper et al., 2020). Notably, the evaluation of CTCs at a single-cell level is usually difficult due to cell rarity and frailty but can be improved by microfluidics or immunoaffinity enrichment methods (Lim et al., 2019). It has also been suggested that the combination of tumor tissue and CTCs samples can better reflect the tumor heterogeneity and offer more clinical information (Figure 2).

In recent years, single-cell sequencing technology has developed rapidly and derived various applications. For example, the Cellular Indexing of Transcriptomes and Epitopes by Sequencing (CITE-seq) enables cell surface epitope and transcriptome measurement at the same time (Stoeckius et al., 2017). Other techniques targeting methylation, histone modification, chromatin accessibility, or the TCR repertoire at a single-cell level provide more precise genomic information for cancer studies. As just mentioned, scRNA-seq and snRNA-seq capture different cellular compositions. The choice between these two technologies depends on sample availability, storage, and biological questions (Slyper et al., 2020). Both technologies allow for sample multiplexing, which largely helps with experiment design and reducing batch effects. More recently, the emerging spatial transcriptomics allows the acquirement of cell transcriptome and cellular position information at the same time, providing informative data for TME studies, like insights into the communication between tumor and immune cells through ligand-receptor interactions at a spatial level.

In Table 1, we list publicly available datasets of TME using different sequencing techniques. Samples can be mouse tumor models or human patient samples. Some studies applied multi-omics techniques for sequencing, including normal single-cell transcriptome, CITE-seq, target V (D)J-seq, and spatial transcriptome analysis. These data are precious resources for us to better understand the underlying immune response and cellular interactions within TME. Although the existence or absence of immune cells can be analyzed through conventional FACS using specific markers, the advantage of single-cell analysis is to perform in-depth transcriptional profiling of immune cells in the tumor samples. With scRNA-seq, individual immune cells could be distinguished and further assist discovering novel subtypes. This helps facilitate our understanding of complicated TME and raise new targets for treatment. Furthermore, the exploitation on multiple scRNA-seq datasets could help define specific gene signatures or specific innate immune cell subsets that respond or resist to cancer immunotherapy, or have predictive or prognostic value in patients undergoing immunotherapy.

**TABLE 1:** Recent publicly available single-cell datasets of TME.

Species and Cell Number	Tumor Type	Sequencing Tech	Data Deposit	Raw Data Availability	Main Findings	Clinical Significance	References
Human, 233,591 cells	Pan-cancer (lung, colon, ovarian, breast cancer)	scRNA-seq, CITE-seq	E-MTAB-8107, E-MTAB-6149, E-MTAB-6653	Yes	BIRC3 as a novel marker of DCs maturation	A pan-cancer blueprint of the heterogeneous TME	Gunaydin, (2021)
					Cancer type-dependent T-/NK-cell prevalence	Monitored dynamic changes in the TME during cancer treatment	
Mouse, 294,912 cells	Syngeneic colon adenocarcinoma (MC38 cell line- immunocompetent mice)	scRNA-seq, spatial transcriptome	GSE164430	Yes	Established a novel spatial transcriptome approach adaptive for multiple z-layers tissue capture	Provided a scalable workflow studying tissue microenvironment, cellular infiltration and interaction	Wu et al. (2021)
Human, 55,832 cells	Cutaneous squamous cell carcinoma (cSCC)	scRNA-seq, spatial transcriptome	GSE144240	Yes	Defined and characterized a tumor-specific keratinocyte cell type in tumor leading edge and related to immunosuppression	Provided single-cell spatial architecture of the inflammatory human cSCC TME	Patel et al. (2018)
Mouse, 17,274 cells	Lung adenocarcinoma (Kras^LSL(lox-stop-lox)-G12D/+^ Trp53^fl/fl^ mouse model)	scRNA-seq, scATAC-seq	GSE134812, GSE145192, GSE151403, GSE145194	Yes	Disruption of RUNX family TFs drive tumor progression and metastasis	A combined gene and motif scores on tumor progression used for survival prediction in human lung adenocarcinoma patients	Jin and Jin, (2020)
Human, more than 200,000 cells	Basal cell carcinoma	scRNA-seq, scATAC-seq	GSE129785	Yes	Discovered regulatory programs controlling T cell exhaustion and a shared program with CD4^+^ T follicular helper cells	Provided a chromatin landscape of intratumoral immunity and immune response after PD-1 blockade	Melaiu et al. (2020)
Human, 208,659 cells	Esophageal squamous-cell carcinoma (ESCC)	scRNA-seq, scV(D)J-seq	GSE160269	Yes	Immunosuppressive ESCC TME and two hidden intermediate phenotypes of fibroblasts	Gene expression levels in the mucosal program prediected ESCC patients survival	Kiss et al. (2020)
Human, 29,825 cells	Melanoma	scRNA-seq, V(D)J-seq	GSE123139; EGAS00001003363	Yes	A wide differentiation spectrum of dysfunctional T cells and a locally induced differentiation process	High level of in CD8 T cells dysfunction is associated with tumor reactivity	Hamm et al. (2016)
Human, 66,627 cells	Nasopharyngeal carcinoma (NPC)	scRNA-seq, scV(D)J-seq	GSE150825	Yes	Prevalence of B cell subpopulations in NPC	Worse progression-free survival in NPC patients with higher proportion of double-negative B cells and MDSCs	Davies et al. (2013)
					Immune-activated and IFN-associated B cells in NPC TME		
Human, 45,000 cells	Breast tumor	scRNA-seq, scV(D)J-seq	GSE114727, GSE114725, GSE114725	Yes	Developed a preprocessing pipeline, SEQC, and a Bayesian clustering and normalization method, Biscuit, to address computational challenges inherent to single-cell data	Supported a model of continuous activation in T cells and do not comport with the macrophage polarization model in cancer	Duan and Luo, (2021)
Human, 560,916 cells	Non-small cell lung cancers after PD-1 blockade	scRNA-seq, scV(D)J-seq	GSE173351, GSE176022	Yes	Expression and reprogramming of mutation-associated neoantigens (MANA) -specific T cells	MANA as important targets of anti-tumor immunity	Xie et al. (2020)
						Selectively upregulation of CD39 in MANA-specific T cells	
Human, 371,223 cells	Colorectal cancer (CRC)	scRNA-seq, spatial transcriptome	GSE178341	Yes	A myeloid-rich inflammatory hub below the colonic lumen in human CRC	Predictions of some multicellular hubs based on transcriptome profile and spatial information	Shaul and Fridlender, (2019)
Human, 77,321 cells	Hepatocellular Carcinoma (HCC)	scRNA-seq	HRA000069, EGAS00001003449	Yes	LAMP3+ DCs are mature conventional DCs with migrating ability to lymph node rather than ascites	Potential HCC biomarkers of ascites-derived myeloid and lymphoid cells	Liu et al. (2021)
Human	Pan-cancer (15 human cancer types)	scRNA-seq	GSE154763	No	LAMP3+ cDCs are broadly present and share different origins among cancer types	SPP1 is a marker for angiogenesis-associated macrophages and linked to poor prognosis	Panda and Colonna, (2019)
Human, 54,285 cells; Mouse	Colon Cancer	scRNA-seq	GSE146771; ENA: PRJEB34105, E-MTAB-8832	No	Two distict TAMs sub-population: C1QC + TAMs involved in phagocytosis and antigen presentation	Anti-CSF1R treatment depleted the inflammatory signature of macrophages but cannot affect pro-angiogenic/tumorigenic gene expression	Bruchard and Ghiringhelli, (2019)
					SPP1+ TAMs for angiogenesis		
Human, ∼ 120,000 cells	Breast tumors, prostate tumors, melanoma	scRNA-seq, CITE-Seq	EGAS00001005115	Yes	Cryopreservation is viable to provide high-quality single-cells for multi-omics analysis	Cryopreservation and sample multiplexing methods for large-scaled projects	Slyper et al. (2020)

High-throughput sequencing techniques offer insights into the intra- and inter-tumoral heterogeneity, tumor origin and evolution, mechanisms of tumor invasion and metastasis, characterization of TME, drug resistance, and future therapeutic design (Li et al., 2021a). The most commonly used computational tools for cancer studies include (Gunaydin, 2021) Seurat and Scanpy, the most commonly used single-cell downstream analysis workflow in R and python platforms (Wolf et al., 2018; Hao et al., 2021); (Wu et al., 2021) Monocle, depicting a “pseudotime” for lineage trajectory prediction (Cao et al., 2019); (Patel et al., 2018) VeloAE, studying cellular transition under different dimension of RNA velocity (Qiao and Huang, 2021); (Jin and Jin, 2020) Cellsnp-lite, genotyping tumor cells at a single-cell level (Huang and Huang, 2021); (Melaiu et al., 2020) MQuad, identifying tumor cell populations with mitochondrial mutations (Kwok et al., 2022); (Kiss et al., 2020) CellChat, inferring cell-cell communication networks and signaling pathways (Jin et al., 2021); (Hamm et al., 2016) FlowGrid, allowing for fast clustering of very large single-cell datasets (Fang and Ho, 2021). Among these, Monocle, VeloAE, and other trajectory analysis tools can help distinguish normal and abnormal differentiation of immune cells, especially for those activated- or pathological-immune cells within tumor sites. They can also help infer the origins of newly discovered immune cell subtypes. On the other hand, Cellsnp-lite and MQuad detect the somatic mutations for tumor clonality identification, separating different tumor clones and the specific immune contexture. CellChat exploits the ligand-receptor expression to depict the crosstalk between innate immune cells and specific tumor clones. For massive tumor datasets analysis, like the pan-cancer analysis, FlowGrid can assist the large datasets combination and clustering within a relatively short time. These robust tools can assist us to analyze immune cell-cancer data from genetic and cellular aspects, providing overall insights into cancer immunology research.

## Role of Innate Immune Cells in the Tumor Microenvironment

### Myeloid Cells

#### Dendritic Cells

Within the TME, DCs function by antigen processing and presentation to T cell surfaces to activate the cytotoxic T lymphocytes (CTLs). Conventionally, DCs can be divided into several subtypes based on their immune phenotype and functions, including CD123^+^ plasmacytoid DC (pDC), CLEC9A^+^ XCR1^+^ CADM1^+^ type1 conventional DC (cDC1), CDA1^+^ CD172A^+^ type2 conventional DC (cDC2), LAMP3^+^ DC, and CD14^+^ monocyte-derived DC (moDC) (Wylie et al., 2019). The single-cell transcriptome profiling on human advanced osteosarcoma and lung cancers in humans and mice provides similar division on DCs subtypes and finds conserved results across two species (Zhou et al., 2020; Zilionis et al., 2019). However, some of the markers that help to differentiate the DC subsets are not transcriptionally enriched but have a good protein expression level. In this scenario, the more recently developed CITE-seq method can better capture the cell surface proteome for a better phenotypical understanding of innate immune cells. In Table 2, we have listed the DCs as well as other innate immune cell transcriptome and cell surface markers to better distinguish relative sub-groups. For example, in one NSCLC (non-small cell lung cancer) scRNA-seq and CITE-seq combined dataset, the authors have also identified these five subtypes of DCs. The antibody-oligo conjugates from CITE-seq provide additional information for cell surface proteome, which is more solid than sole transcriptomic markers (Leader et al., 2021).

**TABLE 2:** Phenotypic markers of innate immune cells using CITE-seq or scRNA-seq. pDC, plasmacytoid DC. cDC1, type1 conventional DC. cDC2, type2 conventional DC. mregDC, mature DC expressing regulatory molecules. moMΦ, monocyte-derived macrophage. AMΦ, alveolar macrophage (tissue-resident macrophage). NSCLC, non-small cell lung cancer. PBMC, peripheral blood mononuclear cells. OVC, ovarian cancer. BAM, border-associated macrophages. CRC, colorectal cancer. Transcriptome markers are mainly derived from clustering results, while surface markers are collected based on antibody-derived tags (ADT) by CITE-seq or FACS antibodies.

Cell Type	Sub-group	Species	Tissue	Transcriptome Markers	Surface Markers	References
Dendritic cells	—	human	Cord blood	—	CD11c, CD14	Lim et al. (2019)
	pDC	human	PBMC	APP, JCHAIN, FAM129C, PPP1R14B	CD4-1, CD305, CD123, CD304	Stoeckius et al. (2017)
	—	human	NSCLC	GZMB	CD123	Li et al. (2021a)
	cDC1	human	NSCLC	CPV1, C1orf54, CLEC9A, IRF8	CD26, CD141	Li et al. (2021a)
	cDC2	human	PBMC	CLEC10A, HLA-DQA1, CD1C	CD1c, Intergrin-7	Stoeckius et al. (2017)
	—	human	NSCLC	ALOX5AP, CLEC10A, FCER1A, CD1A	CD1c, CD5	Li et al. (2021a)
	mregDC	human	NSCLC	BIRC3, CCR7, LAMP3, TXN	CD1c, CD86, PD-L2, CD127	Li et al. (2021a)
	moDC	human	NSCLC	C1QB, C1QA, ALOX5AP	CD16, CD14, CD163, CD40	Li et al. (2021a)
	—	human	OVC ascite	FCN1, S100A9, VCAN, FCGR1A, FCGR1B, CD1C, FCER1A, IFITM2, CLEC10A, FCGR2B	—	Hao et al. (2021)
Monocytes	—	human	Cord blood	—	CD11c, CD14	Lim et al. (2019)
	—	human	NSCLC	C1QA, APOE, FABP4, S100A9, S100A8	CD206, CD33, HLA-DR, CD14, CD141, CD123	Li et al. (2021a)
	—	human	Bone marrow and blood	—	CD14	Wolf et al. (2018)
	—	human	PBMC	—	CD11b	Cao et al. (2019)
	CD14^+^ Mo	human	Bone marrow	CD14	CD14	Qiao and Huang, (2021)
	—	human	PBMC	CD14, S100A8, S100A9	CD64, Folate, CD36, CD11b-1, CD11b-2	Stoeckius et al. (2017)
	CD16^+^ Mo	human	Bone marrow	FCGR3A	CD16	Qiao and Huang, (2021)
	—	human	PBMC	FCGR3A, CDKN1C, TCF7L2	CD16, Folate	Stoeckius et al. (2017)
Macrophages	moMΦ	human	NSCLC	NR1H3, SPP1, MERTK, SIGLEC1	CD206, CD169, CD163, CD40	Li et al. (2021a)
	AMΦ	human	NSCLC	PPARG, SERPINA1, MARCO, VSIG4	CD10, CD206, PD-L2, PD-L1	Li et al. (2021a)
	Microglia	mice	Brain	—	CD45^Low^CD11b^+^CD38^Low^MHC-II^Low^Tmem119^+^Mrc1^–^	Huang and Huang, (2021)
	Microglia	mice	Aged brain	Cx3cr1, Tmem119, P2ry12, Hexb, Cst3	CD45^Low^CD11b^+^	Huang and Huang, (2021)
	BAM	mice	Aged brain	Cd74, Apoe, H2-Aa, H2-Ab1, Mrc1	CD45^Low^CD11b^+^	Huang and Huang, (2021)
Neutrophils	—	mice	Brain	S100a9, S100a8, Retnlg, Lcn2	Ly6C^+^Ly6G^+^	Huang and Huang, (2021)
NK cells	—	human	Cord blood	—	CD56, CD8a, CD16	Lim et al. (2019)
	—	human	NSCLC	GZMB, KLRD1, PRF1, SPON2, GNLY	CD16, CD56, CD45RA	Li et al. (2021a)
	—	human	PBMC	—	CD56	Cao et al. (2019)
	CD56^bright^ CD16^−^ NK	human	Bone marrow	XCL1	CD56	Qiao and Huang, (2021)
	CD56^dim^ CD16^+^ NK	human	Bone marrow	—	CD16	Qiao and Huang, (2021)
ILCs	—	human	PBMC	GATA3, IL1R1, KLRB1, CDC14A, IL7R	CD25, CD45RB	Stoeckius et al. (2017)
	ILC1	human	CRC	CD3D, CD3G, CCL4, IFNG, IKZF3, PRDM1	Lin^−^CD45^+^CD127^+^	Kwok et al. (2022)
	—	human	PBMC	ETS1, TBX21, EOMES, IFNG, BCL11B, TCF7	Lin^−^CD45^+^CD127^+^CD117^−^CRTH2^−^	Jin et al. (2021)
	—	human	Lung, blood, colon and tonsil	IL7R	Lin^−^CD45^+^CD127^+^CD117^−^CRTH2^−^	Fang and Ho, (2021)
	ILC2	human	Lung, blood, colon and tonsil	IL7R, GATA3, MAF, PTGRD2, HPGDS	CD3^−^CD4^−^Lin^−^CD45^+^CD127^+^CD117^+/−^CRTH2^+^	Fang and Ho, (2021)
	ILC3	human	Lung, blood, colon and tonsil	KIT, IL1R1, IL23R, RORC	CD45^+^Lin^−^CD127^+^CD117^+^CRTH2^−^	Fang and Ho, (2021)
	LTi	mice	Fetal liver	Tcf7^+^Zbtb16^−^	Lin^−^IL-7Rα^+^Flt3^-^α4β7^+^CXCR5^+^PLZF^-^	Wylie et al. (2019)

In TME, pDCs mainly secrete type I interferons (IFN-I), which can improve anti-tumor immunity through an interaction with tumor and immune cells. However, their antigen presentation ability is much lower than cDCs and is thought to initiate immune tolerance through the secretion of tolerogenic factors including interleukin 10 (IL-10), tumor growth factor (TGF-β) (Villadangos and Young, 2008). Besides, pDCs express some T cell ligands such as programmed cell death ligand 1 (PD-L1) to bind to inhibitory T cell receptors (Matta et al., 2010). However, whether pDCs correlate with better or poor prognosis in tumors is still controversial. Various studies have pointed out the infiltrating pDCs in tumors with poor prognosis in ovarian cancer and melanoma (Labidi-Galy et al., 2012; Aspord et al., 2013). However, more recent studies have shown that higher densities of tumor-infiltrating pDCs correspond to better survival in triple-negative breast cancer and colon cancer (Oshi et al., 2020; Kießler et al., 2021). A large-sample cohort investigation on breast cancer multi-omics datasets also supported this conclusion (Tian et al., 2021). In nasopharyngeal carcinoma (NPC), the favorable prognostic value of pDCs suggests their anti-tumor effects within TME (Chen et al., 2020a). These conflicting conclusions might be due to different tumor types and can be further confirmed by performing pan-cancer analysis based on abundant publicly available datasets.

cDCs can be divided into two groups, CLEC9A^+^ XCR1^+^ cDC1 requiring BATF3 and IRF8 for maturation, and CD1A^+^ cDC2 dependent on IRF4 (Murphy et al., 2016). cDCs are the most powerful antigen-presenting cells to induce T cell-mediated immune responses through MHC class I cross-presentation and MHC class II presentation in humans. cDCs also infiltrate the tumors but only account for a small portion of immune cells in TME (Böttcher et al., 2018; Diao et al., 2018). In TME, cDC1 plays a key role in launching anti-tumor immunity of CTLs (Roberts et al., 2016). Tumor-associated antigens from tumor sites are transferred to tumor-draining lymph nodes by these cDC1s and followed by CD8^+^ T cells activation (Salmon et al., 2016). cDC1 also regulates the local immune response through chemokines XCL1 and CCL5 produced by NK cells (Böttcher et al., 2018). In single-cell pan-cancer analysis, cDC2 shows variation among different cancer types with diverse functions like development and maintenance of Langerhans cells and promoting T cell differentiation (Cheng et al., 2021). The same study has also conveyed that the CXCL9^+^ cDC2s subtype is more likely to transit into LAMP3^+^ cDC and acquired an enhanced immune-suppressive phenotype.

Notably, one single-cell RNA-seq study on human NSCLC samples has revealed a new type of mature DC expressing regulatory molecules (mregDCs) (Maier et al., 2020). The follow-up studies from the same group further characterize this new subtype with LAMP3 and PD-L1 expression (Leader et al., 2021). In another single-cell transcriptome dataset from human hepatocellular carcinoma, researchers found another small proportion of cDC cells specifically expressing maturation marker LAMP3, migration marker CCR7, lymphocyte recirculation chemokines CCL19 and CCL21 (Zhang et al., 2019). In the single-cell bladder urothelial carcinoma dataset, LAMP3^+^ DCs also exhibit T cell recruitment function, suggesting an immunosuppressive TME (Chen et al., 2020b). In a pan-cancer analysis, researchers further found that cDC1-derived LAMP3^+^ DCs and cDC2-derived LAMP3^+^ DCs are under different ligand-receptor control, suggesting potentially diverse functions (Cheng et al., 2021). This evidence concludes the migrating function of cDCs from tumor to lymph node and the communication with lymphoid cells, suggesting this DC subtype as a potential target for immunotherapy.

Another type of DCs is the moDC, also called inflammatory DC. They only show up in response to inflammation, infection, and cancer (Laoui et al., 2016). A single-cell data on peritoneal ascites from ovarian cancer patients have characterized moDCs and end-stage moDCs. These moDCs use the vacuolar pathway and can efficiently induce cytotoxic CD8^+^ T cells (Tang-Huau et al., 2018). However, in TME, the role of moDCs is still elusive. Some study has shown that they are essential for CD8^+^ T cells and anti-tumor response (Kuhn et al., 2015). In contrast, other studies provided some tumorigenic evidence of moDCs, like inhibitory immune response through nitric oxide release and reactive radical (Laoui et al., 2016).

#### Monocytes

In humans, monocytes can be divided into three main subtypes, CD14^++^ CD16^−^ classical monocytes, CD14^+^ CD16^++^ non-classical monocytes, and CD14^++^ CD16^+^ intermediate monocytes, which have also been validated by several single-cell transcriptome datasets (Dutertre et al., 2019; Zhang et al., 2020). According to tumor patients’ clinical data, alterations of monocytes in peripheral blood are informative for diagnosis and prognosis (Kiss et al., 2020). For example, a higher number of CD14^+^ HLA-DR^low^ monocytes is linked to later cancer stages and poor survival (Okla et al., 2018). More interestingly, the number of CD14^+^ HLA-DR^low^ monocytes progressively drop in patients who received and responded to anti-CTLA4 immune checkpoint blockade, suggesting their prediction value (Gebhardt et al., 2015).

During tumorigenesis, monocytes show various functions at different stages. The classical monocytes can differentiate into pro-tumor TAMs in many tumor types. In a single-cell RNA-seq dataset on human bladder urothelial carcinoma, monocyte-TAMs polarization is observed along with the recruitment of monocytes from normal mucosal tissues (Chen et al., 2020b). In a single-cell pan-cancer analysis, tumor and adjacent normal tissue-derived monocytes express more HLA genes and macrophage-related genes, suggesting a migration of monocytes and maturation into TAMs (Cheng et al., 2021). In human colon cancer, FCN1^+^ monocytes enriched in tumors show a similar pattern to blood CD14^+^ monocytes, probably migrating to tumors and obtaining some tumor-specific transcriptional profiles (Zhang et al., 2020).

In a pan-cancer analysis, tumor-infiltrated monocytes can also promote tumor growth by suppressing T cell functions through some inflammatory cytokines and chemokines (Cheng et al., 2021). According to clinical data, intra-tumoral chemokine CCL2 produced by monocytes are negatively correlated to CD8^+^ T cell numbers within hepatocellular carcinoma patients (Li et al., 2017). In comparison, patients suffering from pancreatic cancer with low CCL2 and high CD8 show a better prognosis (Sanford et al., 2013). Within the TME, they also secrete chemokine CCL5 that enroll immunosuppressive regulatory T cells (Tregs) (Plitas et al., 2016). In the meantime, secretions including IL-4, IL-10, and IL-13 by Tregs are likely to further promote the differentiation of classical monocytes into activated TAMs, progressing the immunosuppression (Pommier et al., 2013). In addition to T cells interactions, monocytes also secrete chemokines CCL3, CCL4, CCL5, and IL-15, which are essential for NK cells recruitment in metastatic tumors sites (Hanna et al., 2015; Kubo et al., 2017).

#### Macrophages

Typically, macrophages can be classified into proinflammatory M1 macrophages and anti-inflammatory M2 macrophages. M1 macrophages can be induced by IFN-γ and lipopolysaccharide (LPS) and can directly attack tumor cells or pathogens and promote inflammatory effects by releasing cytokines like TNF-α, IL-6, IL-12, IL-18 (Mills et al., 2016). They can also present the antigens with MHC class II, activating the adaptive immune responses. In comparison, the M2 are induced by IL-4 or IL-13, and usually have lower MHC class II expression (Lawrence and Natoli, 2011). In TME, the tumor-associated macrophages (TAMs) are more M2-like based on their transcriptome profiling (Qian and Pollard, 2010). However, more and more evidence has shown that the simple division of M1/M2 macrophage in TME is not inclusive, as they found that TAMs share both M1 and M2 patterns (Biswas et al., 2006; Chávez-Galán et al., 2015). Macrophages are of high plasticity and can be affected by the local microenvironment. Many single-cell transcriptome datasets have also pinpointed the inaccuracy of M1 vs. M2 classification (Sathe et al., 2020; Zhang et al., 2020; Zhou et al., 2020; He et al., 2021). In some cancer like pancreatic ductal adenocarcinoma and nasopharyngeal carcinoma, an M1-M2 coupled pattern rather than separate states has been observed in single-cell data, which calls for more precise definitions of TAMs (Peng et al., 2019; Jin et al., 2020). According to single-cell pan-cancer analysis, TAMs subtypes have shown distinct transcriptomic profiles among different cancer types (Cheng et al., 2021). The SPP1^+^ TAMs and C1QC1^+^ TAMs are more like M2 phenotype, while ISG15^+^ TAMs express more M1-related genes. This again emphasizes the limitation of the simple division of M1/M2.

According to a human single-cell colon cancer dataset, C1QC^+^ and SPP1^+^ TAMs are likely to differentiate from FCN1^+^ monocytes in tumors (Zhang et al., 2020). The gene expression profile of C1QC^+^ TAMs is more related to phagocytosis and antigen presentation, while SPP1^+^ TAMs show a preferential expression pattern in angiogenesis regulation, suggesting the potential invasive function of this subtype. In the same study, researchers found that expression of C1QC and high expression of SPP1 correlate to poor prognosis of patients, implying that SPP1 depletion on TAMs might result in a better outcome. In hepatocellular carcinoma, there is another interesting subtype called tissue-resident FOLR2^+^ TAMs, showing similar patterns to fetal liver macrophages and support the oncofetal reprogramming concept in TME (Sharma et al., 2020). These TAMs have more interaction with other immune cells through some immune checkpoints, like CD28^−^CD86, SIRPA-CD47, and CD86-CTLA4. Compared with SPP1^+^ TAMs, FOLR2^+^ TAMs also exhibit more immune-suppressive interactions with Tregs.

**FIGURE 3:**
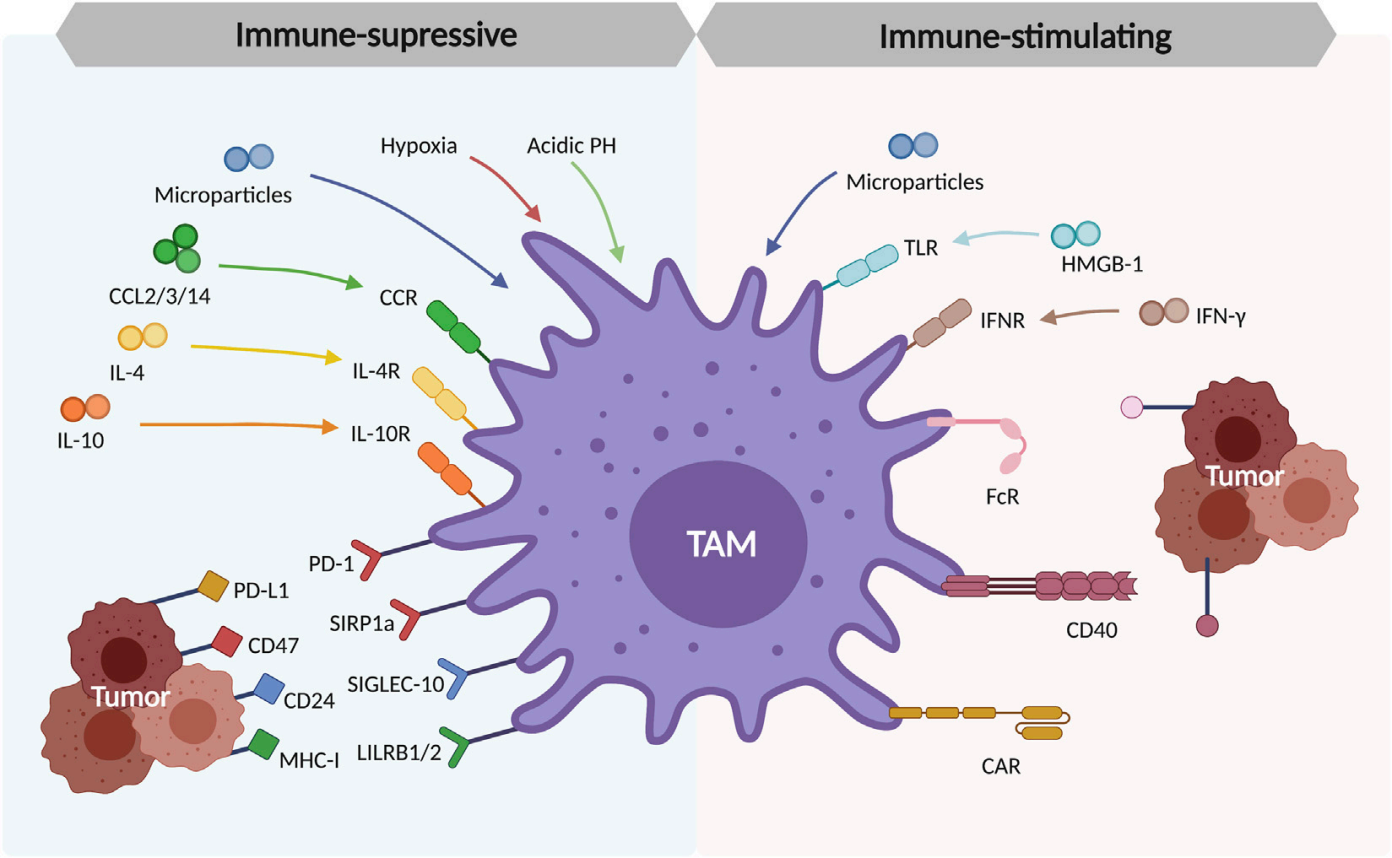
Polarization and formation of tumor-associated macrophages (TAMs) in TME. The tissue-resident macrophages and recruited macrophages from monocytes are the origins of TAMs. These TAMs are polarized in response to the cytokines produced by tumor cells in TME (CCL2/3/14, IL-4, and IL-10), the environmental factors like hypoxia and acidic PH in TME, the immunosuppressive checkpoints (PD-1/PD-L1, CD47/SIRP1a, CD24/SIGLEC-10, MHC-I/LILRB1/2).

In TME, TAMs can promote tumor progression in many aspects. They secrete the chemokines or cytokines like IL-6, IL-8, and IL-10 (Xu et al., 2014; Wang et al., 2021). IL-6 is mainly responsible for cell cycle regulation, tumor angiogenesis, and local inflammation improvement. It can also promote cancer stem cell self-renewal through STAT3 phosphorylation (Wan et al., 2014). In the meantime, IL-8 can activate a wide range of pathways like JAK2/STAT3/Snail pathways, leading to monocytes recruitment and further macrophage polarization (Fu et al., 2015). Notably, IL-8 is highly expressed by TAMs and the serum IL-8 can be used as a predictive biomarker for immune checkpoint blockade efficacy (Schalper et al., 2020). TAMs induce tumorigenesis through several immunosuppressive checkpoints. A famous ligand-receptor pair is programmed cell death protein (PD-1) and programmed cell death-ligand 1 (PD-L1). As reported in a large NSCLC cohort analysis, high expression of PD-L1 in TAMs is correlated to better overall survival (Liu et al., 2020). Other checkpoints include two “don’t eat me signaling”, CD47-SIRPα signaling and CD24-SIGLEC10 signaling, especially expressed on macrophages and tumors (Yu et al., 2016; Barkal et al., 2019). TAMs can also promote cancer metastasis by delivering small cell vesicles called exosomes, packaging the molecules like proteins and nucleic acids. Mass spectrometric analysis has shown the existence of apolipoprotein E (ApoE) within the exosomes, activating the PI3K-AKT pathway and inducing epithelial-mesenchymal transition (EMT), increasing tumor metastasis potential (Zheng et al., 2018) (Figure 3).

On the other hand, TAMs enhance anti-tumor ability through the blockade of some signaling molecules on the tumor cells. Some tumor cells express MHC class I component β2-microglobulin signaling ligand. The knockout of its receptor LILRB1 on macrophages inhibited tumor growth (Barkal et al., 2018). LILRB1 inhibition combined with anti-CD47 monoclonal antibody treatment improved the macrophage phagocytosis and tumor-killing effects, without hurting normal cells (Barkal et al., 2018). Besides, TAMs can function against tumors through macrophage-mediated programmed cell removal. The toll-like receptor (TLR) pathway activated by IL-10 will further induce the BTK signaling pathway and release the “eat me” signaling, which can be a promising immunotherapeutic target (Byrne et al., 2013).

#### Neutrophils

In cancer, the role of the neutrophils is still controversial. These cells are involved in many cancer types, located inside the tumor or surrounding tissues, or in the peripheral blood. In the tumor sites, neutrophils can be stimulated by cytokines and polarized into tumor-associated neutrophils (TAN), with either anti-tumor activity (N1) or a pro-tumor activity (N2), like the nomenclature of M1/M2 macrophages (Shaul and Fridlender, 2019; Veglia et al., 2021). Decades before, scientists have relied on meta-analysis to evaluate the role of neutrophils in cancer, revealing that TANs resided in tumor tissues correlated to unfavorable recurrence-free and poor overall survival (Shen et al., 2014). However, the exact anti-tumor or pro-tumor function of neutrophils in TME is hard to evaluate as there are no accurate markers for distinguishing the populations, which calls for further studies using scRNA-seq and CITE-seq to better characterize them. In one single-cell dataset on human colon tumor samples, researchers have tried Smart-seq2 and 10 × scRNA-seq platform for neutrophils enrichment (Zhang et al., 2020). However, either technique sufficiently captures this cell type, probably due to the difficulty of cell purification procedure and RNA capturing from cells.

There are a group of cells called myeloid-derived suppressor cells (MDSCs), including CD11b^+^Gr1^high^ polymorphonuclear MDSCs (PMN-MDSCs) and CD11b^+^Gr1^low^ (M-MDSCs) in mice. While in human, PMN-MDSCs are often referred to as CD14^−^CD15^+^CD66b^+^CD16^+^CD11b^+^CD33^+^HLA-DR^−^ (Shaul and Fridlender, 2019). These PMN-MDSCs are usually considered pathologic neutrophils due to shared markers with normal neutrophils (Long et al., 2021). However, another review has pointed out that PMN-MDSCs can only stand for a sub-group of neutrophils with divergence at the cell activation level, but not a whole new cell type (Shaul and Fridlender, 2019). With this debate, a recently published single-cell RNA-seq dataset on mice breast tumor models has provided an overview of neutrophils and PMN-MDSCs at the transcriptome level (Alshetaiwi et al., 2020). They have separated the neutrophils from the spleen in homeostasis and MDSCs derived from the MMTV-PyMT mouse breast cancer model and checked how PMN-MDSCs emerge as a distinct cell cluster from neutrophils. Their analysis pointed out that in TME, PMN-MDSCs diverge from neutrophil progenitors and follow an alternative maturation process.

Taken together, although the single-cell transcriptome profiling in mice can provide a better understanding of neutrophil cell fate decisions and developmental trajectory, a protein expression level measurement like CITE-seq could be the best way forward. Another tricky thing is that most of the phenotype characterization and functional tests are conducted in mice models owing to the technical difficulties to enrich neutrophils in human tumor samples. However, neutrophils in these two species are largely different in biology and functions, which calls for the need for more human studies (Masucci et al., 2019). Besides, many experimental studies focus on human circulating neutrophils for functional assessment, which cannot fully mimic the intra-tumoral ones.

### Lymphoid Cells

#### Natural Killer Cells

NK cells can be identified with unique CD56^+^ CD3^−^ markers. Conventionally, NK cells can be divided into two subtypes, CD56^dim^ CD16^+^ NK cells and CD56^bright^ CD16^−^ NK cells. Differences between these two subtypes are that CD56^dim^ CD16^+^ NK cells are the major cluster of NK cells with higher production of cytokines and robust cytotoxic activity, while the latter one is more similar to T helper cells and with less cytotoxic activity (Cooper et al., 2001). Recently, single-cell transcriptome and trajectory analysis revealed that there might be an additional NK cell type residing in bone marrow, NK0 cells, that can differentiate into CD56^dim^ CD16^+^ NK cells and CD56^bright^ CD16^−^ NK cells (Crinier et al., 2021).

In TME, NK cells can directly attack cells with the help of cytokines like IL-12, IL-15, and IL-18. The MHC class I balances activation and inhibition states (Huntington et al., 2007). In addition, they can assist other immune cells like DCs and T cells to launch an anti-tumor response. Studies based on the mice *in vivo* test and single-cell transcriptome analysis on human nasopharyngeal carcinoma have revealed that NK cells recruit cDC1s by secreting chemokines CCL5, XCL1, and XCL2, which also link to the patients’ better overall survival (Böttcher et al., 2018; Gong et al., 2021). In the lung adenocarcinoma murine model, activated NK cells by tumor ligands facilitate the T cell response and lower the tumor progression (Schmidt et al., 2019). However, TME can restrain NK cell functions. For example, the TME-recruited TAMs and Tregs inhibit CD8^+^ T cells and NK cells through cytokines TGF-β, IL-10 and lead to higher expression of inhibitory checkpoints (Ben-Shmuel et al., 2020). As reported by a single-cell study on human melanoma samples, NK cell populations are significantly lower across the patients (de Andrade et al., 2019). In a mass cytometry by time-of-flight (CyTOF) combined with single-cell transcriptomics on the human lung adenocarcinoma dataset, NK cells are the least abundant cells compared with other immune cells (Lavin et al., 2017). These NK cells at tumor sites lowly express CD57 and IFN-γ, while tumor-infiltrated NK cells exhibit higher expression of CXCR3. Another single-cell omics study on nasopharyngeal carcinoma has revealed that they didn’t observe the exhaustion markers like KIR, NKG2A, LAG3, or HAVCR2, and pointed out the NK cells as promising TME-based targets for cancer therapy, as they keep activated and can be a positive regulator in immune response (Gong et al., 2021). Collectively, these findings have suggested that various cancer types might lead to the difference in the frequency of NK cells, their activation status, and the expression of inhibitory markers. In this case, a more thorough analysis of publicly available single-cell datasets can provide more insights into the correlation between the tumor intrinsic factors and innate immune cell phenotypes.

#### Innate Lymphoid Cells

Innate lymphoid cells are another type of highly heterogeneous lymphocyte. They lack the antigen receptors but express diverse activating and inhibiting receptors, responsible for different functions (Artis and Spits, 2015). Based on their dependent transcription factors and cytokines for development, ILCs can be divided into three groups and five cell types, group one containing NK cells and ILC1, group two within ILC2, group three containing ILC3, and lymphoid tissue inducer (LTi) cells. However, it is not that easy to markedly separate these sub-groups because of similar transcriptome profiles and cell state plasticity. Many studies have performed scRNA-seq analysis on mouse or human tissues and obtained some conclusive markers (Ghaedi et al., 2020; Qi et al., 2021). For example, high expression of SLAMF1 in the colon and rectal tumors correlated to much higher survival in patients (Qi et al., 2021). Other phenotypic markers of ILC classification have been well-summarized in another review paper (Roma et al., 2021). The CITE-seq method also showed that these ILCs possess high plasticity in mouse brain and intestinal tissue (Golomb et al., 2020). These cells function differently within TME in promoting and suppressing tumor growth, with a correlation to poor or better clinical outcomes (Yuan et al., 2021).

In group one ILCs, NK cells, and ILC1 lineage diverge due to the required transcription factor T-bet, as ILC1 completely relies on T-bet for its development (Vivier et al., 2018). These different developmental requirements lead to the lower cytotoxic ability of ILC1 and different marker gene expression Tbet^+^ Eomes^−^ (Simonetta et al., 2016). In TME, ILC1, which is similar to T helper cells, can also respond to IL-12 and IL-15 cytokine-enriched environments and produce IFN-γ. However, it is still ambiguous whether ILC1 possesses anti-tumor or pro-tumor effects. The secreted IFN-γ by ILC1 can recruit and activate monocytes and macrophages for phagocytosis function (Panda and Colonna, 2019). On the other hand, ILC1 has shown pro-tumor function and favors tumorigenesis in response to TGF-β (Gao et al., 2017). A recent single-cell transcriptome study on human colorectal cancer has confirmed its transcriptional similarity to T helper cells (Qi et al., 2021). They also detected another cluster of helper-like ILC1 subset with additional expression of TIGIT, which is described as intermediate ILC1s in methylcholanthrene-induced mouse tumor models (Gao et al., 2017). Interestingly, they also observed an ILC1 to ILC3 transition in the blood of cancer patients, but not in normal groups (Qi et al., 2021). However, the underlying mechanism is still unknown and requires further studies.

In cancer, ILC2 has anti-tumor and pro-tumor effects, depending on its cytokine secretion and communication with other immune cells. Clinical data has shown the increase of ILC2 in peripheral blood and tumor tissue in many cancer types, like breast and gastric, but none in the healthy gut samples (Bie et al., 2014; Salimi et al., 2018; Qi et al., 2021). ILC2 produces IL-4 and IL-13, which polarize the macrophages into M2 and participate in pro-tumor activities. Amphiregulin secreted by ILC2 controls the local inflammation through Tregs but also induces immune suppression and promotes the EGFR + tumor growth (Zaiss et al., 2015). However, there are also anti-tumor effects reported. IL-33 activates ILC2 and secrete CXCL1 and CXCL2, as well as the CXCR2 on the tumor surface. This engagement limits tumor growth (Kim et al., 2016).

Group three ILCs contain ILC3 and LTi cells. Single-cell analysis reveals the developmental divergency of ILC3 and LTi cells (Ishizuka et al., 2016). ILC3 is the innate counterpart of Th17, representing both anti- and pro-tumor effects. It has been reported in a human single-cell colorectal cancer dataset that this cancer type won’t affect the heterogeneity of ILC3 subtypes (Qi et al., 2021). According to the diffusion map analysis on single-cell transcriptome data, ILC3 can transdifferentiate into ILCreg under TGF-β signaling stimulation at the late stage of colorectal cancer, which promotes tumor growth (Wang et al., 2020). The secretion of IL-22 by ILC3 also promotes tumorigenesis (Ghaedi and Ohashi, 2020). In contrast, ILC3 restricts the tumor-associated T cell functions with NKp46 expression (Crome et al., 2017). In a mice melanoma model, ILC3 expressing NKp46 prevents tumor growth by helping immune cells infiltrate into tumor sites (Eisenring et al., 2010). For LTi cells, their recruitment to the tumor site is related to CCL21 expression by tumor cells, indicating immune tolerance and invasion (Shields et al., 2010).

## Precise Definitions of Innate Immune Cells With Single-Cell Technology and Its Insight in the Prognosis of Cancer Patients

As mentioned in the previous section, the emergence of single-cell technologies allows precise definitions of innate immune cells during the development and within TME at an overall transcriptome level rather than a single molecule. Furthermore, CITE-seq technology provides a simultaneous evaluation of the transcriptome and the surface proteome of single cells, acting as a high-throughput FACS analysis combined with general single-cell RNA-sequencing. In Table 2, the common innate immune cell transcriptome and cell surface markers allow a better understanding of relative sub-groups and their functions in TME. For example, one scRNA-seq and CITE-seq dataset containing paired tumor and normal lung tissues from 35 NSCLC patients has depicted the immune response of early-stage lung cancer (Leader et al., 2021). Notably, CITE-seq data with Antibody-Derived Tags (ADTs) can offer supplementary information for cellular classification as high-dimensional models. These integrated immune phenotypes defined by scRNA-seq and CITE-seq further determine an immune activation signature that is related to immune checkpoint blockade rather than overall immune content alterations.

The advance of single-cell technology came to the stage where we could predict the prognosis of cancer patients through the precise definition of innate immune cells in the tumor microenvironment. The conventional definition of innate immune cells depends on a limited number of markers including surface proteins and cytokines, mainly with flow cytometry. With the increase of subtypes of innate immune cells, increasing overlap of markers between different cell populations makes definition challenging. For instance, IL-17 production is one of the key features of ILC3. However, IL-17 is expressed in both ILC3 and LTi cells, requires additional markers to distinguish them (Borger et al., 2019). Heterogeneity adds up more complexity. A subtype of ILC3 labeled with NCR produces IFN-γ rather than IL-17 (Pantazi and Powell, 2019). Compelling evidence demonstrates that the use of scRNA-seq broadens available markers to define innate immune cells more precisely than flow cytometry.

The use of scRNA-seq discovered novel functions of innate immune cells. For instance, Trem2 + macrophages associate with adipocytes and regulate lipid metabolism, indicating the role of macrophages as metabolic sensors as well as potential therapeutic targets in metabolic diseases (Jaitin et al., 2019). Single-cell technology can also facilitate our understanding of the intracellular gene network and crosstalk among various cellular components in the complicated tumor immune landscape. As mentioned above, CCL5 secreted by NK cells recruits cDC1 and assists the anti-tumor function. Expression of CCL5 in NK cells also correlates with better overall survival in melanoma patients (Mgrditchian et al., 2017). However, CCL5 produced by monocytes recruit immunosuppressive Tregs, which can induce themselves into activated TAMs and progress the immunosuppression. These complicated molecular and cellular networks are hard to evaluate by conventional technologies but can be depicted by higher resolution sequencing methods like scRNA-seq and scATAC-seq.

The precise definition of innate immune cells matters in the understanding of the tumor microenvironment. TAMs have been classically defined into M1 (pro-inflammatory) and M2 (anti-inflammatory) based on the expression of CD80, CD206. However, recent data from scRNA-seq of renal cell carcinoma demonstrated TAMs could be separated into more than 5 distinct populations based on the expression of immune checkpoint genes. TAMS of patients who responded to immune checkpoint blockades exhibited more pro-inflammatory phenotypes as well as an increase of co-inhibitory genes such as VSIR, VSIG4, PD-L2, and SIGLEC10 compared with non-responders (Bi et al., 2021). The observation indicates the potential prognostic value of scRNA-seq data in cancer patients. ILCs have been classified based on flow cytometry and the expression of key transcription factors (Spits et al., 2013). Although the definition has been standard in the field of immunology, increasing evidence suggests that ILCs could adapt their fate based on the tumor microenvironment. In the lung cancer enriched with IL-23, ILC1s were converted to ILC3s. The shift from tumor-killing ILC1s to tumor-promoting ILC3s was reflected in the poor prognosis of the patients’ cohort with the high level of IL-23 expression and the increase of tumor-promoting ILC3s in the tumor (Koh et al., 2019). A recent study with scRNA-seq of metastatic pancreatic cancer cells in the liver revealed that pancreatic cancer cells adopt the intermediate transcriptional state between the pancreas and liver. Tumor microenvironment signals such as TGF-β drove the transcriptional heterogeneity and drug response. *In vitro* organoid models that lack immune cells could not recapitulate the transcriptional heterogeneity observed *in vivo* (Raghavan et al., 2021). Not only the prognosis prediction value but also this study indicates the significance of immune cells in the tumor microenvironment to shape the phenotype of cancer cells. Indeed, TGF-β, causative to the transcriptional heterogeneity in this study, is one of the major immune-suppressive cytokines that potentially explains the organoid models lacking immune cells did not recapitulate the change (Pickup et al., 2013). With these observations, we anticipate that single-cell technology will enable the precision medicine of cancer patients with the precise definition of innate immune cells in the tumor microenvironment.

At the same time, an adequate single-cell experimental design is required to obtain in-depth immune characterization and exclude confounding factors from phenotypic correlations. The choice of technology should be based on the biomedical hypothesis. For example, the combination of single-cell transcriptomics and proteomics provides a better profile of the molecular targets and cell types. The single-cell and spatial transcriptomics aim to find novel genome-wide discoveries at a spatial level. In this way, the pathologists can play a critical role in region selection to cover higher tumor heterogeneity. Large cohort single-cell datasets can be useful for pan-cancer integrative analysis and biomarker discovery through various machine learning models to predict the response to immunotherapy and survival rate. In all, full efforts from experimental, statistical, and computational scientists are required for better study design and data mining, thus realizing the ultimate goal of translational medicine for cancer therapy.

## How to Exploit Innate Immune Cells for Cancer Immunotherapy

Single-cell RNA transcriptome technology has depicted the precise gene expression landscape of TME. Various computational tools such as ligand-receptor mapping and cytokine signaling activity analysis have contributed to the understanding of innate immune cells and their functions in TME (Browaeys et al., 2020; Jiang et al., 2021). Based on these findings, we propose two main directions for immunotherapy strategies for innate immune cells.

The first is the blockade of immune checkpoints on immune cells or tumor cells. Immune checkpoints are initially for self-tolerance to avoid self-attack. However, this has been exploited by some tumor cells to evade immune response (Pardoll, 2012). As innate immune cells exhibit a quicker response to tumors than the adaptive immune cells, they are thought to have a better response to immunotherapy. At the same time, immune cells of myeloid lineages are more infiltrated into the tumors than lymphoid cells. Some pre-clinical and clinical studies have employed this checkpoint blockade concept on myeloid cells for cancer immunotherapy, including the CD47-SIRPα signaling axis, one of the hottest myeloid checkpoints in the past decade. Studies have pointed out the broad expression of CD47 on many solid tumor cell surfaces, including glioblastoma, breast, liver, colon, bladder, ovarian, and prostate cancers (Willingham et al., 2012). Anti-CD47 antibody blockade exhibits inhibitory effects on tumor growth and tumor metastasis in murine ovarian cancer model (Willingham et al., 2012). In phase I and II clinical trials, anti-CD47 antibody or SIRPαFc treatment has shown safety, tolerability, and efficacy in advanced colorectal cancer, non-Hodgkin’s lymphoma for cancer immunotherapy (Feng et al., 2019). In recent years, more and more myeloid immune checkpoints have been discovered, including CD24-SIGLEC10, MHC class I-LILRB1, and CD73^−^CD39 (Barkal et al., 2018; Barkal et al., 2019; Goswami et al., 2020). These promising checkpoints have paved a bright way for cancer immunotherapy.

Other attempts can be the agonists of innate immune responses. Our immune system has many common pathways in response to infection and tumors, including TLR, RIG-I-like receptors (RLRs), and cGAS-STING (Li et al., 2021b). Synthetic molecules designed to mimic these pathways provoke innate immune reactions at the tumor sites independent from tumor antigens. For example, TLR agonists can initiate both innate and adaptive immune systems and exhibit anti-tumor effects in *vivo* mouse models (Davis et al., 2011). Another reported small-molecule agonist targeting TLR-2 can suppress leukemia progression through cytotoxic T cell activation (Cen et al., 2019). Similarly, STING agonists have also been shown to have anti-tumor effects in the mouse melanoma model (Demaria et al., 2015). This anti-tumor immunity was further elevated through PD1 and CTLA4 blockade. Many clinical trials for agonist monotherapy and combined treatment with immune checkpoint inhibitors have been conducted in solid tumors and lymphoma (Jiang et al., 2020). However, the more in-depth research is required as the innate immune system can be a double-edged sword to the downstream initiation of adaptive immunity. For example, STING signaling can activate the innate myeloid cells, but can also cause T cell stress and Treg infiltration through indoleamine 2,3-dioxygenase (Liang et al., 2015). Thus, further studies are required for the investigation of the whole cGAS-STING cascade for future safer immunotherapy.

## Conclusion

Innate immune cells are the first barrel of our immunology. The innate immune cells directly attack the tumor cells, while acting as a messenger to bridge the innate immune and adaptive immune system together for anti-or pro-tumor effects. In this review, we summarize key cell types in the innate immune systems and their functions in TME. We referred to the recently published single-cell omics dataset to present a precise functional conclusion at a single-cell level. We provide current and prospective biomarkers for predicting patient survival or treatment effect on immunotherapy. We also conclude the promising immunotherapy targets for further studies. As TME is a complicated structure comprising various environmental properties and cellular communication, an overall understanding of TME offers us insights into new immunotherapy investigation.

## Data Availability

The datasets presented in this study can be found in online repositories. The names of the repository/repositories and accession number(s) can be found in the article in Table 1.
